# Surgeon Involvement in Pre-Clinical Medical Education: Attitudes of Directors of Education

**Published:** 2012-03-31

**Authors:** Simon Turner, Brendan Diederichs, Christopher de Gara

**Affiliations:** 1 Department of Surgery, University of Alberta, Edmonton, Alberta, Canada; 2 Department of Diagnostic Imaging, University of Calgary, Calgary, Alberta, Canada

## Abstract

**Background:**

Application rates to surgical residencies have shown a downward trend recently. Introducing students to surgeons early in medical school can increase interest in surgery as a career and enhance the instruction of important surgical topics. Directors of undergraduate medical education have unique insight and influence regarding the participation of surgeons in pre-clinical education.

**Methods:**

To understand the attitudes of these educators towards surgeons as teachers in pre-clinical programs, a survey was administered to the directors of undergraduate medical education at each of the English-language medical schools in Canada.

**Results:**

Educators estimate the participation of surgeons in all categories of pre-clinical education to be low, despite being valuable, and think that it should be increased. The most significant barrier to participation identified was a lack of surgeons’ time.

**Conclusions:**

Despite the value of surgeons participating in pre-clinical education, their rate of participation is low. Steps should be taken to facilitate the involvement of surgeons in this phase of education, which may lead to improved education for students and increased student interest in surgery residencies.

## Introduction

Surgeons play a vital role within the healthcare team. Despite the advent of less invasive and non-surgical techniques to treat previously surgical diseases, the need for surgeons to deliver care to patients is likely to endure for the foreseeable future. However, several reports have identified a decrease in medical students applying to surgical residencies in Canada^1^ and the United States,^2^ especially General Surgery. This decline has been attributed to several factors, including students’ concern for their future lifestyle^3^ and the increasing focus of medical schools on training primary care physicians.^4^ The lack of interest in surgery may be most pronounced in the pre-clinical years. One study of UK pre-clinical students found that less than half were willing to even consider a career in surgery.^5^ Similarly, an American study found that only 7% of the pre-clinical students participating were interested in surgery, while 40% of students stated that their interest in surgery increased during clerkship.^6^

The low interest in surgery amongst medical students may be related to limited access to positive role models in surgery in the pre-clinical years. Several studies have demonstrated that early interaction with surgeons can increase students’ interest in surgery as a career^7^,^8^ and can also improve their confidence and feeling of readiness for a surgical clerkship.^9^ Thus, the involvement of surgeons in undergraduate medical education, in particular in pre-clinical medical education, is an important issue. This study approaches this issue by examining the attitudes of Canadian directors of undergraduate medical education towards the involvement of surgeons in pre-clinical education. Directors of undergraduate medical education were chosen for this study because they have unique insight into factors that influence surgeons’ participation in this phase of education, and their attitudes may directly impact the rates of involvement of surgeons in teaching.

## Methods

To study the attitudes of medical educators towards the involvement of surgeons in pre-clinical education, we conducted an online survey in 2010. The survey was distributed to the directors of undergraduate medical education at each of Canada’s fourteen English-language medical schools. The survey included questions that addressed surgeons’ involvement in several aspects of pre-clinical medical education, including lectures, small-group teaching, administration, and student counseling. Respondents were asked to estimate the rate of participation by surgeons, comment on whether that rate was increasing or decreasing with time, whether it should be increased or decreased, and to compare the participation rate of surgeons to that of non-surgeons. The survey also asked respondents to identify barriers to surgeons teaching in pre-clinical education.

## Results

Eight directors of undergraduate medical education responded to the survey. This yields a response rate of 57.1%.

Half of the respondents did not think that surgeons were responsible for teaching the majority of lectures on topics pertaining to surgical illness, and none thought that in topic areas where surgery is applicable, the number of surgeon lecturers was comparable to medical physician lecturers. Only one respondent agreed that the risks, benefit, rationale and operative techniques for specific surgeries were included when covering diseases with surgical treatment options.

Only one respondent disagreed that there is an educational benefit to having surgeons teach about surgical illness. However, all respondents except one estimated a rate of less than 25% when asked about the percentage of medical education taught by surgeons, the percentage of preclinical educators who are surgeons, the percentage of pre-clinical lectures taught by surgeons, the percentage of pre-clinical small-group learning taught by surgeons, the percentage of administrative positions in undergraduate medical education held by surgeons, and the percentage of pre-clinical student counseling done by surgeons. For each category of involvement, the majority of respondents thought that the rate of surgeons’ participation was less than that of non-surgeons and was not changing with time. A majority also stated that surgeons’ involvement in preclinical undergraduate education should be increased (75%). When asked to rate potential barriers to increasing surgeon involvement at their institution on a Likert scale of 1 (not significant at all) to 5 (very significant), the factor rated as the most significant barrier was lack of surgeons’ time (Mean = 4.3). The barriers rated as least significant were lack of administrator interest in surgeons’ teaching (Mean = 2.1) and lack of available teaching spots (Mean = 2.0).

## Discussion

There are multiple benefits of involving surgeons in the education of pre-clinical medical students. At the most basic level, surgeons have the most experience and knowledge to inform the instruction of students about surgical diseases and treatments. Indeed, the majority of respondents to our survey believed that there was an educational benefit to surgeons teaching about surgical illness. In addition, interaction with surgeons can introduce students to the idea of surgery as a career. Furthermore, as has been shown by Kozar et al., “positive encounters with surgeons can favorably influence the perceptions of first-year medical students towards a career in surgery”, possibly helping to combat the declining application rates for surgical residencies.^7:166^

However, our findings reveal that surgeons participate at a low rate in pre-clinical undergraduate medical education. In all of the categories studied, 25% or less of undergraduate education was provided by surgeons. Even in the specific topic areas where surgery is most applicable, surgeon involvement was seen as less than that of non-surgeons. Only one respondent indicated that important aspects of specific surgeries, including the risks and rationale of surgery, were taught when relevant. Overall, this situation represents a detriment to the education of medical students. Not only are students not being introduced to many surgeons who might serve as role models, but they are potentially not receiving optimal instruction when it comes to surgical diseases and treatments. Whether students become surgeons or not, it is important for all physicians to have at least a basic understanding of surgical diseases and treatments as these are often relevant to any medical practice.

For their part, the directors of undergraduate medical education surveyed did not identify themselves as barriers to surgeons participating in pre-clinical teaching. In fact, the majority thought that surgeon participation should be increased. The barrier that they perceived to be most significant in impeding surgeons from teaching was a lack of time in the surgeons’ schedule available for teaching. Future studies are needed to examine what surgeons identify as barriers to their participation in pre-clinical education to see if their attitudes agree with those of the educators in this study. However, it seems likely that mechanisms that increase the availability of surgeons to teach, such as funded, protected time for educational activities, may increase the participation of surgeons in pre-clinical education.

## Conclusions

Despite the apparent importance of surgeons as pre-clinical teachers, this study demonstrates that their rate of participation in many aspects of pre-clinical education is low. Directors of undergraduate medical education in Canada view the participation of surgeons in this phase of education as valuable, and believe that it should be increased. Efforts should be made to facilitate their teaching in the pre-clinical phase. Doing so has the potential to improve medical student education, and may have an impact on students’ interest in surgery as a career.

The major limitation of this study is its small sample size. However, while the target population of this study is small, this group of educators is highly knowledgeable and influential in Canadian medical education. As such, this population is an important one to examine, and was, therefore, selected for this study. Future studies are needed to examine what surgeons identify as barriers to their participation in pre-clinical education to see if their attitudes agree with the findings of this study.
